# Interplay Between MicroRNAs and Breast Cancer Therapies: Personalized Therapeutic Potential for HER2-Low Breast Cancer

**DOI:** 10.3390/cancers17223672

**Published:** 2025-11-16

**Authors:** Eduarda Carvalho, Fernando Schmitt, Nuno Vale

**Affiliations:** 1 PerMed Research Group, RISE-Health, Faculty of Medicine, University of Porto, Alameda Professor Hernâni Monteiro, 4200-319 Porto, Portugal; ecferreira@med.up.pt (E.C.); fschmitt@med.up.pt (F.S.); 2 RISE-Health, Department of Pathology, Faculty of Medicine, University of Porto, Alameda Professor Hernâni Monteiro, 4200-319 Porto, Portugal; 3 RISE-Health, Department of Community Medicine, Health Information and Decision (MEDCIDS), Faculty of Medicine, University of Porto, Rua Doutor Plácido da Costa, 4200-450 Porto, Portugal

**Keywords:** breast cancer, HER2-low, microRNAs, therapies, precision medicine

## Abstract

HER2-low breast cancer has been recognized as a heterogenous group of tumors influenced by hormone receptor (HR) expression, giving rise to tumors with either a luminal-like phenotype or features resembling triple-negative breast cancer (TNBC). Despite the development of HER2-targeted therapies, several studies have demonstrated their limited efficacy in patients diagnosed with HER2-low breast cancer. However, recent research has led to the development of antibody-drug conjugates (ADCs), such as trastuzumab emtansine (T-DM1) and trastuzumab deruxtecan (T-DXd), with the latter showing promising results in treating these patients. Despite this breakthrough, the availability of a single effective therapy fails to account for tumor heterogeneity and may contribute to the emergence of therapy resistance, leaving HER2-low patients without treatment options. MicroRNAs (miRNAs) are small non-coding RNAs that regulate gene expression at a post-transcriptional level and are capable of modulating key cellular processes. Recent studies have highlighted their potential as therapeutic targets, contributing to the advancement of personalized, patient-center therapies. In this context, the interplay between miRNAs and HER2-targeted therapies, particularly their modulation of common essential genes and signaling pathways, could reshape HER2-low therapy strategies to transform current practices aimed at improving the overall patient outcomes. Therefore, this review aims to elucidate the mechanisms underlying current HER2-targeted therapy and explore a potential crosstalk with miRNAs, ultimately serving as a guide for the development of personalized therapeutic strategies.

## Simple Summary

Breast cancer is not a single disease, but a collection of different tumor types. One molecular subgroup, called HER2-low breast cancer, is especially challenging because it does not respond well to most existing HER2-targeted treatments. To date, patients mostly rely on one therapy, which may not work for everyone and can lead to resistance. This review explores the role of microRNAs, small molecules that regulate gene expression, as cancer biomarkers and therapeutic targets to explain how these tumors behave and respond to treatment. By understanding how microRNAs interact with HER2 and hormone pathways, we hope to elucidate the mechanisms underlying HER2-targeted therapies to explore a crosstalk with microRNAs and design combination therapies that are more precise and effective. This approach could improve patient outcomes and also guide the research community towards more personalized treatment strategies for breast cancer.

## 1. Introduction

Breast cancer is the most predominant cancer worldwide and represents the primary cause of cancer-related deaths among women [1]. Traditionally, breast cancer classification has been based on invasiveness and tissue of origin; however, such classification does not fully reflect the complexity of tumor biology [2]. As a result, molecular classification has emerged to better categorize breast tumors according to hormone receptor (HR) expression and human epidermal growth factor receptor 2 (HER2) status, defining four main molecular subtypes—luminal A, luminal B, HER2-enriched and triple-negative breast cancer (TNBC) [3,4].

HER2 is a transmembrane tyrosine kinase receptor that belongs to the epidermal growth factor receptor (EGFR) family, encoded by the *ErbB2* gene located on the short arm of chromosome 17 (17p) [5]. Unlike other EGFR family members, HER2 activation is not ligand-dependent and occurs through homodimerization or heterodimerization, particularly with HER3. HER2/HER3 dimerization induces a conformational change on HER2, triggering its activation and subsequent downstream signaling pathways, such as phosphatidylinositol-3-kinase/activated protein kinase B (PI3K/Akt) and mitogen-activated protein kinase (MAPK), which promote cell survival, proliferation and differentiation [5,6,7]. In normal tissue, phosphorylated and dephosphorylated forms of HER2 are similarly expressed, maintaining tissue homeostasis and leading to normal breast tissue development. However, in approximately 20% of breast cancer cases, HER2 is overexpressed, leading to constitutive activation, uncontrolled cell proliferation, migration and invasion, being frequently associated with high recurrence rates and poor prognosis [5,8,9,10].

According to the 2023 American Society of Clinical Oncology and the College of American Pathologists (ASCO/CAP) guidelines for HER2 testing, HER2-enriched breast cancer is divided into three subgroups based on immunohistochemistry (IHC) and in situ hybridization (ISH) results. HER2-positive is defined as IHC 3+ or 2+ with gene amplification (ISH positive), HER2-low is characterized by an IHC score of 1+ or 2+ without gene amplification (ISH negative) and HER2-negative, also known as HER2-zero, as IHC 0 [11,12]. HER2-low breast cancer accounts for 55% of all breast cancer cases and has attracted increasing attention within the scientific community regarding whether it constitutes a distinct molecular and clinical entity [13,14,15,16]. Current evidence indicates that HER2-low tumors are heterogeneous, with some exhibiting luminal-like characteristics and others resembling TNBC, influenced by HR expression. Nonetheless, most HER2-low tumors exhibit a luminal-like phenotype [13,17]. In line with this, molecular profiling studies show that tumors characterized by HER2 IHC 1+ and 2+ scores demonstrate increased luminal-like features, whereas HER2 IHC 0 tumors exhibit basal-like characteristics, but these differences lose significance after accounting for HR expression, confirming that HER2-low biology is largely driven by HR expression [13,18]. Consequently, the 2023 ASCO/CAP guidelines conclude that there is insufficient proof to consider HER2-low breast cancer as a distinct biological entity. Consistently, the European Society for Medical Oncology (ESMO) recognizes HER2-low as a heterogenous group of tumors influenced by HR status and states that current data do not support its classification as a separate breast cancer subtype [11,19]. Therefore, this lack of a distinct classification is largely attributable to the incomplete understanding of the molecular mechanisms driving heterogeneity.

Before HER2-low breast cancer was clinically recognized, patients within this subgroup were typically managed according to HER2-positive or HER2-negative protocols, which often resulted in suboptimal therapeutic benefit and unnecessary toxicities due to inadequate target engagement or overtreatment [20]. In the context of breast cancer, tyrosine kinase inhibitors (TKIs) such as lapatinib, neratinib, tucatinib and pyrotinib target the tyrosine kinase domain of the HER2 receptor to block its activity. These agents are primarily used as second- or third-line treatments for HER2-positive breast cancer often in combination with monoclonal antibodies such as trastuzumab to overcome therapy resistance [21,22]. When administered as monotherapy, TKIs have failed to demonstrate clinical benefit in patients diagnosed with HER2-low breast cancer, but later research, particularly the phase III DESTINYBreast-04 trial, revealed that administration of the an antibody–drug conjugate (ADC), trastuzumab deruxtecan (T-DXd), significantly increased progression-free survival (PFS) and overall survival (OS) in this population, leading to its approval for the treatment of this disease [18,20,23]. Despite this clinical advancement, major challenges persist, including an inability to account for molecular diversity, limited therapeutic options beyond T-DXd, the frequent emergence of therapy resistance and potential therapy-related toxicity, underscoring the urgent need for novel molecular insights and precision therapies for this subgroup.

In this context, microRNAs (miRNAs) have emerged as critical post-transcriptional regulators capable of fine-tuning gene expression. These small non-coding RNAs influence a wide range of biological processes, including developmental timing, embryogenesis, cell differentiation, organogenesis, metabolism and apoptosis, as well as in the pathogenesis of numerous diseases [24,25]. In cancer, aberrant miRNA expression contributes to tumorigenesis by disrupting key regulatory networks. Oncogenic miRNAs are typically overexpressed, whereas tumor suppressor miRNAs are downregulated in disease, leading to the activation of proliferative signaling, uncontrolled cell growth, enhanced migration and invasion, angiogenesis and resistance to apoptosis. Notably, the same miRNA may act as an oncogene in certain pathologies and as a tumor suppressor in others, depending on the cellular environment and target genes [26,27,28]. Given their broad regulatory capacity, miRNAs have been shown to modulate the expression of HR and HER2 signaling components, thereby influencing treatment response. For example, miR-342 overexpression is significantly associated with ER-positive and HER2-positive breast tumors [29], while miR-18a downregulation correlates with increased ESR1 expression in ER-positive tumors [30]. Such findings suggest that miRNAs may contribute to the heterogenous expression of HR observed in HER2-low breast cancer, potentially distinguishing HR-positive from HR-negative tumors.

MiRNA-based therapeutic strategies, including miRNA mimics and inhibitors, aim to restore normal expression patterns by enhancing tumor suppressor miRNAs or suppressing the function of oncogenic miRNAs that contribute to pathological conditions [24,31]. These tools can inhibit the upregulation of oncogenic miRNAs, thereby allowing the expression of their tumor suppressor targets, and restore the downregulation of tumor suppressor miRNAs to inhibit their target oncogenes [32]. While the therapeutic potential of miRNAs has been validated in multiple cancer types, including HER2-positive and TNBC [33,34,35,36,37], their role in HER2-low tumors remains largely unexplored. Investigating how miRNAs modulate HER2 and HR signaling could provide critical insights into the molecular basis of tumor heterogeneity and therapy resistance, ultimately informing the development of more effective and personalized strategies for patients with HER2-low breast cancer.

## 2. Molecular and Genomic Landscape of HER2-Low Breast Cancer

HER2-low breast cancer comprises a heterogenous group of tumors in which HR is a key determinant of the molecular profile. Multiple transcriptomic and genomic studies, often stratified by HR status, have revealed distinct patterns between HER2-low/HR-positive and HER2-low/HR-negative tumors, with implications for both disease biology and therapeutic strategies.

Comprehensive molecular analyses of tumors are fundamental for understanding cancer biology, guiding treatment decisions and improving patient outcomes. Several studies employing PAM50 analysis on HER2-low tumors have indicated that luminal-related gene expression is predominantly linked to tumors classified as IHC 2+ and HR-positive, while IHC 0 and HR-negative tumors show a higher prevalence of genes associated with TNBC subtype. Moreover, these analyses revealed that HER2-low/HR-positive tumors are primarily classified within the luminal subtype, whereas HER-low/HR-negative tumors tend to cluster within the TNBC subtype [15,17,38]. These findings support tumor stratification based on HR status, confirming its role as a key driver of heterogeneity within HER2-low breast cancer, with the majority of cases being HR-positive [39,40]. From a clinicopathological perspective, HER2-low tumors are more frequently of no special type (NST), regardless of HR status. HER2-low/HR-positive tumors show a lower proliferation index compared to HER2-low/HR-negative tumors, displaying a negative correlation between lower Ki-67 levels and HR expression, while HER2-low/HR-negative tumors present elevated Ki-67 indices [41,42,43]. This pattern is consistent with luminal tumors, which typically have lower Ki-67 levels, and TNBC tumors, which display higher Ki-67 levels [44]. Therefore, distinguishing HER2-low/HR-positive from HER2-low/HR-negative tumors at the transcriptomic and genomic levels are crucial for understanding the underlying biological mechanisms that lead to this divergence.

Understanding the mutational landscape of HER2-low breast cancer may aid in treatment decisions. While investigating the genomic characteristics of this subgroup, Taratino et al. [45] reported that the most prevalent oncogenic mutation in ER-negative tumors was TP53 (79.8%), whereas PIK3CA (38.2%), GATA3 (16.1%) and ESR1 (13.4%) mutations were more frequently observed in ER-positive tumors. The same four genes were altered under similar conditions in HER2-negative samples. When comparing the two HER2 status groups and adjusting for ER expression, mTOR mutations were enriched in HER2-low samples, whereas MAP3K1 and TP53 mutations were more common in HER2-negative tumors. However, after correction for multiple hypotheses testing, no statistically significant differences remained between genomic landscapes of HER2-low and HER2-negative samples. Consistently, Bansal et al. [46] found that ESR1 (14.3%) and PIK3CA (42.5%) mutations were more common in HER2-low/HR-positive tumors compared with HER2-low/HR-negative, while TP53 mutations were significantly more prevalent in HER2-low/HR-negative (74.3%) than in HER2-low/HR-positive (25%). When comparing HER2-low/HR-negative with TNBC, they found no differences in ESR1 mutation frequency, but observed significantly more PIK3CA mutations in HER2-low/HR-negative tumors and more TP53 mutations in TNBC. Similarly, Juan et al. [47], using next-generation sequencing on 445 HER2-low breast cancer samples, showed that although not statistically significant, PIK3CA mutations were more frequent in HR-positive tumors, while PTEN mutations appeared more common in HR-negative tumors. In contrast, ESR1 and TP53 mutations were significantly enriched in HR-positive and HR-negative tumors, respectively. Furthermore, an analysis on germline mutations revealed that BRCA1 alterations were more prevalent in HR-negative tumors, while BRCA2 mutations were more frequently detected in HR-positive tumors.

Assessing copy number variations (CNV) of *ErbB2* gene can help elucidate whether HER2 expression is influenced by gene dosage or by other regulatory mechanisms. Tan et al. [48] performed a bioinformatics analysis using the TCGA-BRCA and METABRIC databases to explore the influence of ErbB2 CNVs on ErbB2 mRNA expression and found that elevated mRNA levels corresponded to higher CNV scores in HER2-low breast cancer, with a positive but weak correlation between HER2 IHC scores and CNV scores. When stratified by HR status, the same trend was observed, but in the HR-negative subgroup, the correlation between HER2 IHC scores and CNV scores was not statistically significant. These findings suggest that although ErbB2 CNVs contribute to HER2 protein expression in HER2-low/HR-positive tumors, other mechanisms are likely responsible for modulating HER2 expression in HER2-low/HR-negative tumors. Likewise, another study [49] compared CNV values across HER2-positive, HER2-low and HER2-negative samples, observing similar CNV values between HER2-low and HER2-negative tumors, but significantly higher CNV values in HER2-positive compared to HER2-low and HER2-negative, an indication of gene amplification. The lack of difference between HER2-low and HER2-negative suggests that their disparity is not driven by gene dosage, but rather by other regulatory mechanisms. Altogether, these findings support the notion that HER2-low breast cancer should not be considered a distinct molecular entity, but rather a group of heterogenous tumors with either luminal-like features or TNBC-like characteristics, modulated by HR expression.

## 3. MicroRNAs’ Biogenesis and Mechanism of Action

Small non-coding RNA molecules with an average length of 21–22 nucleotides are termed miRNAs. These molecules are important regulators of gene expression at the post-transcriptional level and also play roles in numerous cellular processes, including cell differentiation, proliferation and survival [24,50].

**Figure 1:**
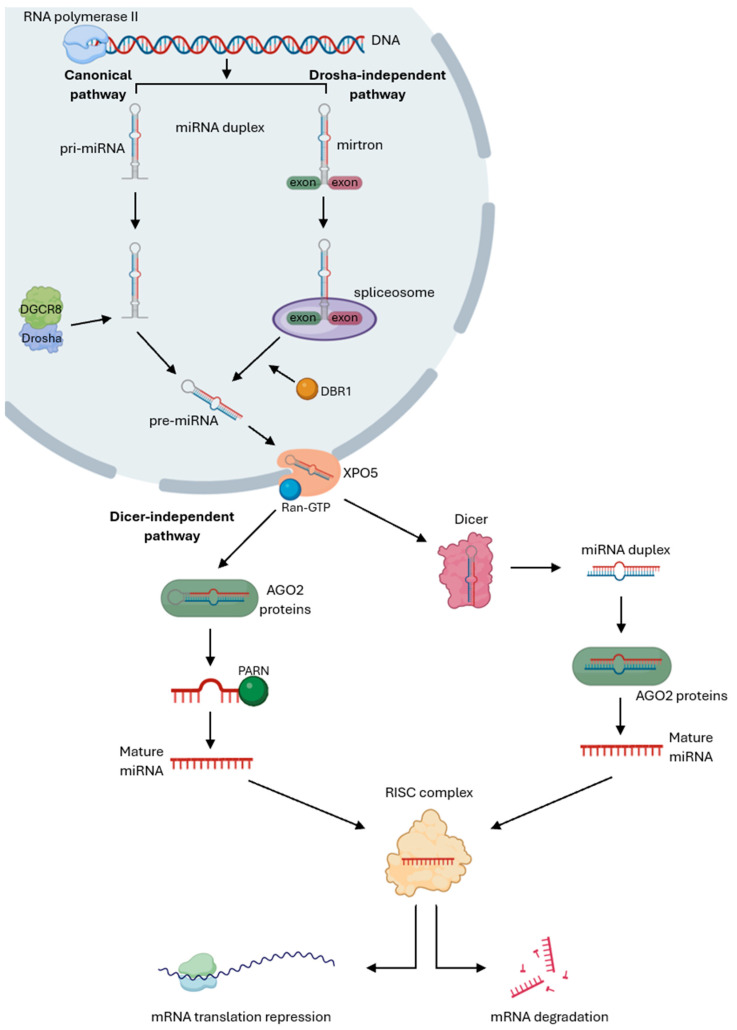
Canonical and non-canonical pathways of miRNA’s biogenesis. Canonical pathway starts with RNA polymerase II transcribing a specific gene into a primary miRNA (pri-miRNA), which is cleaved by the Drosha/DGCR8 Microprocessor complex into a precursor miRNA (pre-miRNA). This pre-miRNA is exported to the cytoplasm by the Exportin 5 (XPO5)/Ran-GTP complex to be processed by Dicer to produce a miRNA duplex, which is loaded with AGO2 proteins and degraded by cellular machinery. The guide strand is incorporated into the miRNA-induced silencing complex (miRISC) to guide the complex to their mRNA targets and regulate translation repression or mRNA degradation. The Drosha/DGCR8-independent pathway starts with mirtrons, which are spliced out by the spliceosome and linearized by the DBR1 debranching enzyme, producing a debranched pre-miRNA that is exported to the cytoplasm by the XPO5/Ran-GTP complex and processed by Dicer, followed by miRNA maturation through the same cytoplasmic steps as in the canonical pathway. The Dicer-independent mechanism starts with the pri-miRNA being cleaved by Drosha to form a pre-miRNA that is directly loaded with AGO2 proteins to be processed into a pre-miRNA intermediate that is trimmed at the 3’-end by PARN, producing a single-stranded mature miRNA. The mature miRNA then follows the same cytoplasmic steps as the canonical pathway to repress mRNA translation or promote mRNA degradation [Created in Biorender. Eduarda Carvalho. (2025) https://app.biorender.com/].

The biogenesis of miRNAs can follow two different processing mechanisms: canonical and non-canonical as illustrated in Figure 1. The canonical pathway begins with RNA polymerase II transcribing a specific DNA segment in the nucleus into a long primary miRNA (pri-miRNA), which folds into a hairpin structure. The Microprocessor complex, composed of the RNase III enzyme Drosha and the RNA-binding protein DiGeorge Syndrome Critical Region 8 (DGCR8), then cleaves the pri-miRNA into a stem-loop precursor miRNA (pre-miRNA), which is exported to the cytoplasm by the Exportin 5 (XPO5)/Ran-GTP complex. In the cytoplasm, the pre-miRNA is processed by Dicer, another RNase III enzyme, which removes the loop region to produce a miRNA duplex consisting of the 3p strand, called the passenger strand, and the 5p strand, called the guide strand. The passenger strand is loaded with Argonaute proteins, AGO2, and degraded by cellular machinery, while the guide strand, due to its AGO selectivity and lower thermodynamic stability, is incorporated into the miRNA-induced silencing complex (miRISC) to guide the complex to the 3’ untranslated region (UTR) of their mRNA targets and regulate translation repression or mRNA degradation and deadenylation through base-pair complementarity [24,50,51,52].

Conversely, the non-canonical pathway omits some steps of the canonical mechanism and can be Drosha/DGCR8-independent or Dicer-independent. The Drosha/DGCR8-independent process originates from short hairpin introns, called mirtrons, which are spliced out by the spliceosome instead of being processed by the Microprocessor complex. The splicing generates an intron lariat, that is linearized by the debranching enzyme DBR1, producing a debranched pre-miRNA that is exported to the cytoplasm by the XPO5/Ran-GTP complex and processed by Dicer, followed by miRNA maturation through the same cytoplasmic steps as in the canonical pathway. In the Dicer-independent mechanism, the pri-miRNA cleaved by Drosha forms a short hairpin pre-miRNA that is too short for Dicer processing. Instead, AGO2 proteins directly process this hairpin to generate a 30-nucleotide long fragment, which is further cleaved by the Poly(A)-specific ribonuclease (PARN) to yield a mature miRNA. The mature miRNA is subsequently incorporated into the RISC complex to mediate gene silencing [53,54,55,56].

Mature miRNAs typically bind to the 3’ UTR of target mRNAs through perfect or partial base-pair complementarity, leading to degradation or translation repression [57,58]. Although less common, miRNAs can also interact with 5’ UTR, exerting either repressive or activating effects on translation [59]. For instance, miR-10 enhances ribosomal proteins production by binding to their 5’ UTR, while other studies [60,61] have reported translation repression through 5’ UTR binding, highlighting an additional regulatory layer that increases miRNAs versatility. In addition to these post-transcriptional roles, miRNAs can act as endogenous decoys that bind to transcription factors or interact directly with promoter regions of protein-coding genes, resulting in transcriptional activation or repression [62,63,64].

The function of miRNAs is further determined by subcellular localization [65]. Although enriched in the cytoplasm, miRNAs are also found in the nucleus, where they can regulate transcription through promoter or transcription factor interactions. MiR-30b-5p overexpression targets the 3’ UTR of ASPP2 to suppress its expression and activate p-Akt signaling, which inhibits apoptosis and promotes invasion in breast cancer [66]. Additionally, downregulation of miR-542-3p increases p-Akt levels, reducing FOXO1 recruitment and leading to transcriptional suppression [67]. In HER2-positive breast cancer cells, miR-21 inhibition increases PTEN expression and contributes to trastuzumab resistance [68]. Furthermore, a subset of miRNAs, known as mitomiRs, are localized in the mitochondria where they modulate mitochondrial gene expression and cellular metabolism [69,70]. In addition, miRNAs are also present in extracellular compartments such as exosomes, microvesicles, apoptotic bodies and protein complexes, which can be detected in various body fluids including serum, plasma, saliva, urine, breast milk, cerebrospinal fluid, and other biological fluids [71,72]. Encapsulation protects miRNAs from degradation by ribonucleases, ensuring stability, cellular uptake and facilitating cell-to-cell communication, making them valuable diagnostic and prognostic biomarkers for a wide range of diseases [72,73]. In this context, Otmani et al. [74] reported that exosome-derived miR-24-3p promotes T-cell apoptosis in leukemia via DENN/MADD regulation, and Zhao et al. [75] demonstrated that exosome-derived miR-934 from colorectal cancer cells enhances the PI3K/Akt signaling pathway, promoting M2 macrophage polarization.

Taken together, these findings underscore miRNAs as key post-transcriptional regulators of gene expression in both intracellular and extracellular environments. In the context of HER2-low breast cancer, understanding miRNA biogenesis and function is particularly relevant, as their dysregulation may contribute to heterogeneity.

## 4. MicroRNAs in Cancer

MiRNAs can function as either oncogenes or tumor suppressors. In carcinogenic conditions, oncogenic miRNAs are frequently upregulated, whereas tumor suppressive miRNAs tend to be downregulated. By regulating key signaling pathways, these small molecules contribute to the activation of cancer hallmarks and play a central role in tumor initiation and progression [27]. Notably, a single miRNA can simultaneously regulate multiple hallmarks by targeting distinct pathways involved in different aspects of carcinogenesis. This versatility makes miRNAs not only important for understanding cancer biology, but also promising as disease biomarkers. As biomarkers, they can provide diagnostic, prognostic and predictive information that reflects molecular alterations that may vary across specific cancer cell subpopulations [76,77]. Furthermore, because a single miRNA can target numerous mRNAs, its effects may be paradoxical, thereby increasing the therapeutic response in one context while contributing for disease progression and therapy resistance in another [28]. Therefore, elucidating the multiple roles of miRNAs in cancer is essential for exploring their potential as biomarkers and therapeutic agents.

### 4.1. Function of MicroRNAs in Cancer Hallmarks

In 2000, Hanahan and Weinberg [78] proposed six hallmarks that normal cells must acquire to enable tumor development and metastasis, serving as a basis to understand the mechanisms of cancer and the broad spectrum of malignant behaviors. With advances in cancer research, additional hallmarks were identified, leading to the current list, which includes sustained proliferative signaling, evading growth suppressors, resisting cell death, enabling replicative immortality, inducing angiogenesis, activating invasion and metastases, reprogramming of energy metabolism, evading immune destruction, genome instability and mutation and tumor-promoting inflammation [79].

Although cancer hallmarks are described as distinct capabilities, they are highly interconnected and often reinforce one another during tumor progression, as shown in Table 1. Sustained proliferative signaling arises when cancer cells overexpress oncogenes that drive growth pathways and evade growth suppressors, creating an optimal cellular environment for malignant expansion to maintain continuous cell division, as cancer cells skip regulatory checkpoints that normally restrain cell division, thereby leading to uncontrolled proliferation [79,80,81]. For instance, the upregulation of miR-96-5p in renal cell carcinoma downregulates PTEN expression and increases cell proliferation [82], while the upregulation of miR-449a downregulates mutant p53 expression, which decreases cell proliferation in breast cancer, through inactivation of the PI3K/Akt pathway [83]. At the same time, genome instability and mutations facilitates abnormal cells survival rather than inducing apoptosis, as they have acquired advantageous traits to resist to cell death [84,85]. Furthermore, to maintain long-term growth, cancer cells achieve replicative immortality through reactivation of telomerase reserve transcriptase (TERT), which elongates telomeres by adding repetitive sequences to chromosome ends [86,87]. However, rapid proliferation of cancer cells often surpasses their vascular supply causing hypoxia, which promotes the transcription of several pro-angiogenic genes, including vascular endothelial growth factor (VEGF) to induce angiogenesis and facilitate invasion and metastases by offering new routes for tumor cell dissemination [88,89,90]. Nevertheless, invasion requires epithelial tumor cells to lose E-cadherin and increase N-cadherin and vimentin expression to enable cells to undergo epithelial-to-mesenchymal transition (EMT). In parallel, cancer cells secrete metalloproteinases to degrade extracellular matrix (ECM), thereby facilitating migration and invasion [91,92]. In melanoma, upregulation of miR-155-5p suppresses SOCS1 expression, leading to JAK2/STAT3 pathway activation and overexpression of matrix metalloproteinase-9 (MMP9), VEGFa and FGF2 factors, ultimately enhancing angiogenesis, proliferation and migration [93] and in ovarian cancer, upregulation of miR-29c-3p suppresses migration and invasion by directly targeting MMP2 [94]. Simultaneously, cancer cells are reprogramming their energy metabolism, shifting from oxidative metabolism to aerobic glycolysis (Warburg effect), increasing the synthesis of biomolecules to fuel proliferation and increasing the energy necessary for invasion [95,96]. Lactic acid, a byproduct of glycolysis, accumulates in tumor microenvironment (TME) and causes extracellular acidification, thereby promoting the polarization of tumor-associated macrophages (TAM) towards an M2-like phenotype, which in turn secrete immunosuppressive and pro-angiogenic factors such as transforming growth factor-beta (TGF-β), interleukin-6 (IL-6) and VEGF. These mediators facilitate immune evasion by suppressing anti-tumor immune responses, while simultaneously stimulating angiogenesis and tumor progression [97,98]. In breast cancer, upregulation of miR-155 increases IL-6 expression, which contributes to hexokinase 2 enhancement via activation of the STAT3 pathway, leading to increase glucose metabolism [99], while miR-155-5p increases M2 macrophage polarization, contributing to active immune response in pancreatic cancer [100]. Therefore, alterations in one hallmark tend to affect others producing heterogeneous tumors.

### 4.2. MicroRNAs as Biomarkers in Cancer

In addition to being expressed in tumor tissues, miRNAs are also found in various biological fluids, where they are referred to as circulating miRNAs. These circulating molecules can be secreted by tumor cells or released as a consequence of apoptosis and they exhibit remarkable stability in these fluids against endogenous ribonucleases, temperature fluctuations and pH variations due to their encapsulation within exosomes, microvesicles, apoptotic bodies or their association with protein complexes [71,72]. Importantly, miRNAs display tumor-specific signatures that reflect cancer development and progression, providing valuable information for accurate diagnosis and prognostic evaluation regarding a specific treatment [123].

Diagnostic biomarkers detect or confirm the presence of a certain pathology or condition, thereby supporting disease classification [124]. For instance, miR-21 is a well-established cancer biomarker, as its upregulation correlates with breast [125], lung [126], colorectal [127] and gastric [128] cancers, enabling clear distinction between healthy individuals and patients. Additionally, miR-21 is strongly associated with poor survival and disease recurrence, also serving as a prognostic and predictive biomarker [129,130,131]. A prognostic biomarker provides information about the probability of disease recurrence or progression in patients with an existing condition, while predictive biomarkers identify patients who are more likely to respond positively or negatively to a therapy or environmental factor [124]. Elevated plasma levels of miR-141 can serve as a diagnostic biomarker, with 87.8% sensitivity and 80.0% specificity for detecting gallbladder cancer, and are also associated with poor survival [132]. Increased expression of miR-210 in hypoxic exosomes derived from bone marrow-derived mesenchymal stem cells promotes invasion of lung cancer cells and metastasis, making this miRNA a diagnostic biomarker of hypoxia-driven lung cancer and high co-expression of miR-210 in cancer and stromal cells is a positive prognostic biomarker for disease-specific survival [133,134]. Additionally, increased expression of miR-10b serves as a prognostic biomarker for disease progression and metastasis in breast cancer [135], while downregulation of serum miR-218 in gastric cancer is linked to disease progression, metastasis and poor OS [136,137]. Similarly, reduced miR-126 expression predicts distant metastases, high tumor grade and poor survival in colorectal cancer [138]. Furthermore, miR-31 acts as a predictive biomarker associated with reduced PFS and OS in patients receiving anti-EGFR therapies, as well as a prognostic biomarker for disease recurrence [139,140]. High serum miR-125b expression correlates with advanced disease stage and chemotherapy resistance, suggested by elevated proliferation and reduced apoptosis, serving as a predictive biomarker of poor clinical outcome [141] and the cluster miR-221/222 predicts disease recurrence and tamoxifen resistance in breast cancer, correlating with treatment failure [142]. Altogether, these examples highlight the potential roles of specific circulating miRNAs as non-invasive diagnostic, prognostic and predictive biomarkers, underscoring their clinical relevance for improving disease detection, patient stratification and therapeutic decision-making.

However, tumors are inherently heterogeneous, consisting of diverse subpopulations of cells with distinct genetic, transcriptomic and phenotypic profiles. Intra-tumor heterogeneity reflects the differences among cancer cells within the same tumor and inter-tumor heterogeneity encompasses variations in the same cancer type between different individuals [143]. Additional factors, such as socioeconomic status, demographic region, nutrition and lifestyle behavior, can influence miRNA expression at the cellular level, potentially increasing tumor heterogeneity and affecting disease diagnosis [144,145]. This complexity is often masked by conventional molecular analysis techniques, which typically evaluate bulk samples and provide only average measurements of miRNA expressions, indicating misleading results. Consequently, single-cell analysis has emerged as a powerful approach to unravel tumor heterogeneity and provide deeper insights into cancer biology [146,147,148]. Moreover, current therapeutic strategies based on bulk analysis fail to account for distinct subclones within the same tumor and while treatment may suppress certain populations, it can simultaneously favor the survival of more aggressive or therapy-resistant cells, ultimately aggravating disease progression [149,150]. Therefore, employing single-cell analysis to investigate the molecular and genomic characteristics of HER2-low tumors and to identify differently expressed miRNAs may help tailor patient-specific therapeutic approaches with better clinical outcomes.

## 5. Targeted Therapies for Breast Cancer

Typically, breast cancer therapy involves a combination of surgery, neoadjuvant and adjuvant treatments. However, advances in the understanding of breast cancer biology have provided deeper insights into the genomic, transcriptomic and proteomic alterations underlying this disease. Consequently, therapeutic research is shifting toward patient-specific approaches, leading to the development of targeted therapies tailored to each patient, with the aim of improving outcomes and turning conventional broad-spectrum therapies increasingly obsolete [151,152].

In line with this, T-DXd remains the only agent that has demonstrated clinical efficacy in patients with HER2-low breast cancer [20]. Nevertheless, reliance on a single therapy fails to address tumor heterogeneity, which may effectively eliminate sensitive cancer cell populations and simultaneously foster the persistence of resistant clones. Furthermore, it may increase mutational pressure on the target, thereby facilitating the emergence of therapy resistance [153,154]. Therefore, understanding the mechanisms underlying HER2-targeted therapies aligned with the understanding of the molecular and cellular pathways involved in HER2-low breast cancer heterogeneity, is essential for the development of novel therapeutic strategies targeting HER2 and HR to delay disease progression.

### 5.1. HER2-Targeted Therapies in Breast Cancer

**Figure 2:**
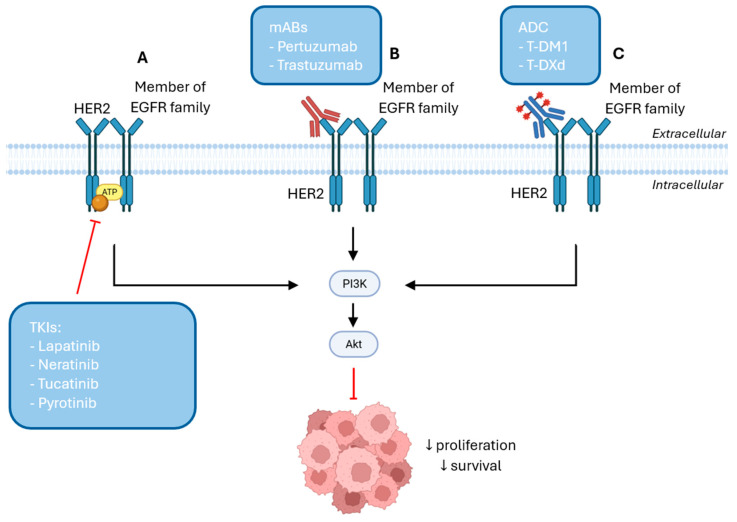
(**A**) TKIs bind to the ATP-binding pocket and inhibit kinase activity, blocking PI3K/Akt signaling pathway, further reducing cell survival and proliferation. (**B**) mABs bind to HER2 on the tumor cell surface and either promote receptor internalization or inhibit its dimerization with other members of the EGFR family, inhibiting the activation of PI3K/Akt signaling pathway. (**C**) ADCs bind to HER2 on the tumor cell surface and promote their internalization into the cell to release the cytotoxic payload to exert its cytotoxic effect and inhibit cell proliferation and survival by inactivating the PI3K/Akt pathway [Created in Biorender. Eduarda Carvalho. (2025) https://app.biorender.com/].

HER2-targeted therapies include TKIs, monoclonal antibodies (mAB) and ADC, as illustrated in Figure 2 [155]. TKIs are small molecules that target HER2 overexpression and include lapatinib, neratinib, tucatinib and pyrotinib to inhibit the activation of PI3K/Akt and MAPK signaling pathways by competing with ATP for binding to the tyrosine kinase domain of HER2, which prevents cancer progression [156,157,158]. Based on their mechanism of action, TKIs can be categorized into five subtypes. Type I inhibitors compete reversibly with ATP at the kinase domain, inhibiting activity, but exhibiting low selectively, which can result in off-target effects by blocking other kinases. Type II inhibitors also prevent ATP binding in a reversible manner, but preferentially bind to the inactive conformation of their target kinase, providing higher intrinsic selectivity. Type III inhibitors bind to an allosteric side outside the ATP-binding pocket, inducing a conformational change that makes them highly selective. Additionally, type IV inhibitors interact with the substrate-binding site, competing with substrate, which confers high selectivity, and type V inhibitors bind simultaneously to two different sites on the kinase, combining mechanisms to enhance specificity and inhibition [156,159].

Briefly, lapatinib is a reversible, dual type I TKI that blocks HER1 and HER2 by competing with ATP for binding to their kinase domain, inhibiting downstream signaling pathways [160]. Neratinib is an irreversible type I TKI that covalently binds to the ATP-binding pocket of EGFR, HER2 and HER4, which prevents autophosphorylation and blocks the downstream signaling cascades [161]. Tucatinib is a recent reversible TKI that binds to the ATP-binding site of the HER2 kinase domain, selectively inhibiting HER2 heterodimerization with HER3 [162]. Pyrotinib is an irreversible TKI that targets the kinase domains of HER1, HER2 and HER4 to induce cell cycle arrest and suppress tumor growth [157]. Clinical studies have demonstrated that combination therapy with neratinib plus capecitabine improves PFS and OS in patients with HER2-positive metastatic breast cancer, resulting in greater clinical benefit compared with lapatinib plus capecitabine treatment [163]. Similarly, treatment of pyrotinib plus capecitabine resulted in superior PFS and OS compared with lapatinib plus capecitabine, enhancing the clinical benefit for patients with HER2-positive metastatic breast cancer [164]. Moreover, a separate study showed that pyrotinib monotherapy was effective in luminal/HER2-low (IHC 2+, ISH negative) and luminal/HER2-positive breast cancers, with enhanced efficacy when combined with chemotherapy, but no benefit was observed in luminal/HER2-low (IHC 1+) [165].

Moreover, mABs target the extracellular domain of HER2, with trastuzumab and pertuzumab being the most widely used agents. These antibodies inhibit both homodimerization and heterodimerization of HER2, consequently blocking key downstream signaling pathways [166,167]. mABs represent effective therapeutic strategies due to their high target selectivity, stability in circulation and low toxicity. These unconjugated mABs bind to HER2 on the tumor cell surface and induce immune-mediated responses that result in apoptosis [168]. Trastuzumab binds to domain IV of HER2 by electrostatic and hydrophobic interactions, promoting receptor internalization, preventing dimerization and activating the antibody-dependent cellular cytotoxicity (ADCC) mechanism to kill HER2-overexpressing cancer cells [166,169]. Additionally, trastuzumab can also inhibit cell proliferation and induce apoptosis by blocking PI3K/Akt signaling pathway and inhibit VEGF to decrease cell invasion by inactivating the same pathway and thus reduce tumor growth [170]. Pertuzumab, in turn, binds to domain II of HER2 to block dimerization with other HER family members and also recruit immune cells via ADCC [171,172]. In clinical practice, mAB-based therapy is often administrated in combination with chemotherapy, achieving remarkable improvements in PFS and OS [173,174,175]. Also, neratinib either as monotherapy or in combination with trastuzumab showed a significant reduction in HER2-low (IHC 2+, ISH negative) viability, with the combination proving more effective through enhanced inactivation of EGFR, HER3 and p-Akt [176]. Additionally, conjugated mABs, known as ADC, represent an effective therapeutic strategy that combines the specificity of mABs with the potency of cytotoxic payload. After binding to HER2 on the tumor cell surface, ADCs are internalized and transported to lysossomes, where the linker between the antibody and the drug is degraded, releasing the cytotoxic payload to exert its cytotoxic effect [177]. Currently, there are two approved ADC for the treatment of HER2-positive breast cancer, trastuzumab emtansine (T-DM1) and T-DXd [178]. T-DM1 delivers the maytansine DM1 to inhibit microtubule formation, induce cell cycle arrest and cause apoptosis, while T-DXd carries the topoisomerase I inhibitor deruxtecan to inhibit DNA replication and induce cell cycle arrest and apoptosis [177,179,180]. Comparative studies evaluating these ADCs as monotherapies highlight a greater clinical benefit in patients with HER2-positive breast cancer treated with T-DXd [181,182,183]. Similarly, in HER2-low patients, treatment with T-DXd significantly improved PFS and OS compared with physician’s choice (PFS: 9.9 months vs. 5.1 months; OS: 23.4 months vs. 16.8 months), leading to the approval of T-DXd as the first therapy for this patient population [20].

Understanding the mechanism by which HER2 is amplified in HER2-low breast cancer, may help in the development of new therapeutic strategies with already-approved therapies for HER2-positive breast cancer.

### 5.2. HR-Targeted Therapies in Breast Cancer

As most HER2-low breast tumors are predominantly HR-positive [42], understanding the molecular framework of HR-targeted therapies may support the development of new therapeutic strategies for HER2-low.

**Figure 3:**
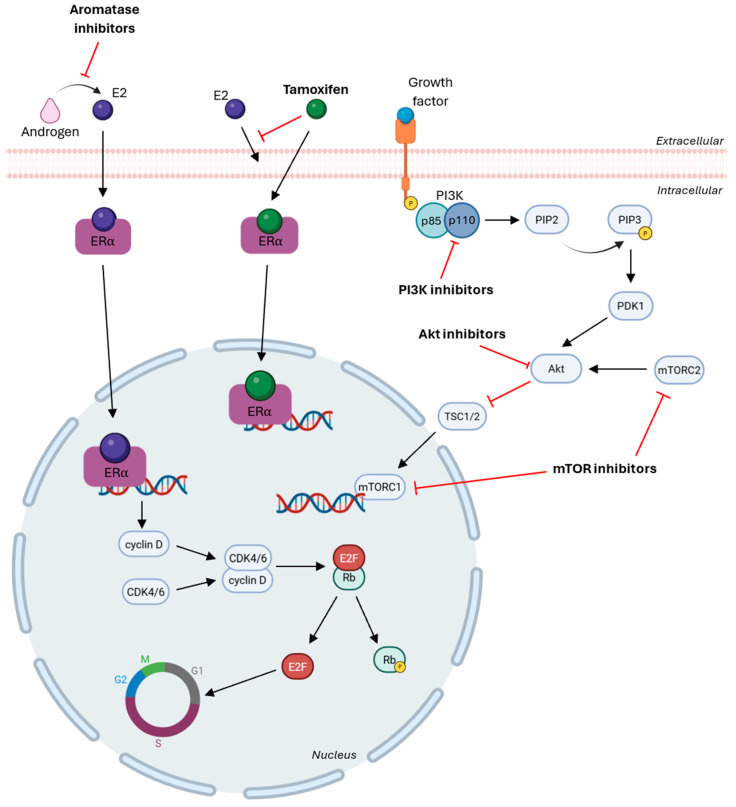
Aromatase inhibitors block androgen conversion into estrogen and tamoxifen binds to ERα to inhibit the transcription of estrogen-responsive elements, blocking cell proliferation. PI3K inhibitors target the kinase domain of an isoform to block its activity in phosphorylation PIP2 into PIP3, AKT inhibitors bind to the ATP-binding pocket to induce apoptosis and mTOR inhibitors block mTORC1 and/or mTORC2 to reduce proliferation. CDK4/6 inhibitors inhibit Rb phosphorylation and stop the progression of cell cycle [Created in Biorender. Eduarda Carvalho. (2025) https://app.biorender.com/].

HR-targeted therapies may be classified as selective estrogen receptor modulators (SERMs) or as aromatase inhibitors (AI), as depicted in Figure 3. SERMs act as estrogen antagonists in breast cancer by competing with estrogen for binding to estrogen receptors (ERs) on the cell surface, thereby interfering with ER signaling and downstream pathways [184]. The most widely used SERM in HR-positive breast cancer is tamoxifen, which competes with estrogen for binding to ERα. This prevents ERα-mediated transcription of estrogen-responsive genes, which inhibits tumor cell proliferation by suppressing the PI3K/Akt/mTOR signaling pathway [185,186]. AIs are available across three generations, with the third generation being the most specific and associated with fewer adverse effects and includes exemestane, anastrozole and letrozole [187]. These therapeutic agents inhibit the aromatase enzyme, which is responsible for the conversion of androgens to estrogens, thereby reducing estrogen production and subsequent ER activation, ultimately inhibiting cell proliferation [188,189]. Comparative clinical trials highlight the therapeutic potential of AIs over tamoxifen, reducing the risk of recurrence in ER-positive breast cancer patients [190,191,192].

Another important therapeutic approach in ER-positive breast cancer resides on targeting critical regulators of cell cycle progression and includes CDK4/6 inhibitors and PI3K pathway inhibitors [193], as shown in Figure 3. Cell cycle progression is controlled by cyclin-dependent kinases (CDK) and cyclins, which contribute to cellular development by interacting and forming CDK-cyclin complexes. These complexes promote retinoblastoma (Rb) phosphorylation, leading to the release of E2F transcription factors and subsequent transcription of genes essential for cell cycle progression [194,195]. In cancer, deregulation of this process results in uncontrolled proliferation and transmission of DNA damage. Therefore, CDK4/6 inhibitors act by binding to CDK4 and CDK6 to prevent the CDK-D cyclin complex formation, which blocks Rb phosphorylation, halts cell cycle progression at the G1 phase, and prevents transcription of genes required for proliferation [196,197].

The PI3K/Akt/mTOR signaling pathway is among the most frequently dysregulated pathways across cancers, contributing to tumor growth, survival and therapy resistance [193]. Upon activation, PI3K is recruited to the plasma membrane, where its regulatory subunit p85 stabilizes and attracts the catalytic subunit p110, which phosphorylates phosphatidylinositol-4, 5-biphosphate (PIP2) to generate phosphatidylinositol-3, 4, 5-triphosphate (PIP3). PIP3 recruits PDK1, which together with mTORC2 facilitates Akt phosphorylation and activated Akt inhibits tuberous sclerosis complex (TSC1/2), leading to activation of mTORC1, resulting in reduced apoptosis and increased proliferation and survival [198,199,200]. There are two main classes of PI3K inhibitors: pan-PI3K inhibitors and isoform-specific PI3K inhibitors. Pan-PI3K inhibitors target the kinase activity of all four isoforms of class I PI3Ks, but have low specificity and are associated with elevated toxicity and off-target effects. By contrast, isoform-specific PI3K inhibitors demonstrate higher efficacy and reduced adverse events by selectively inhibiting individual isoforms and most isoform-specific inhibitors are directed against the p110α catalytic subunit, which is frequently mutated in cancer by PIK3CA mutations [201,202,203]. Additional therapeutic agents include Akt inhibitors and mTOR inhibitors, which also act within the PI3K/Akt/mTOR signaling pathway. Akt inhibitors can be classified as allosteric inhibitors, which block Akt activity by preventing conformational changes and induce apoptosis, or as ATP-competitive inhibitors, which bind to the ATP-binding pocket within the kinase domain of Akt and other AGC family kinases [204,205,206]. mTOR inhibitors are divided into two main groups: allosteric mTOR inhibitors, which bind to FK506 binding protein 12 (FKBP12) of mTORC1 and allosterically inhibit its kinase activity and ATP-competitive mTOR inhibitors, which target the kinase domain of mTOR, inhibiting both mTOCR1 and mTORC2 signaling [204].

A clinical trial [207] evaluating the administration of capivasertib (an Akt inhibitor) in combination with fulvestrant in patients with ER-positive, HER2-negative breast cancer, with or without prior or ongoing treatment with CDK4/6 inhibitors, demonstrated a significant improvement in PFS. Additional research [208] supports the combined use of CDK4/6 inhibitors with PI3K/Akt/mTOR pathway inhibitors, highlighting this strategy as a means to further improve clinical outcomes in these patients. However, combined treatment with CDK4/6 inhibitors plus endocrine therapy results in worse PFS and OS for HER2-low patients compared with HER2-zero, both HR-positive [209,210]. Additional studies [211,212] confirmed that, among HR-positive patients receiving this therapeutic combination, those with HER2-low tumors presented inferior PFS and OS. Multivariable analyses further identified HER2 status as an independent predictor of worse outcomes, suggesting a potential need to target HER2 signaling to prevent progression in HER2-low disease.

## 6. Crosstalk Between MicroRNAs and Breast Cancer Therapies

MiRNAs can regulate key cellular processes and their aberrant expression in carcinogenesis depict their potential to be enrolled in the development of personalized therapeutic strategies. Many studies have reported their therapeutic efficacy in numerous cancers, including breast [213], prostate [214], lung [215], gastric [216], hepatocellular carcinoma [217] and many others.

### 6.1. Advances and Challenges in MicroRNA-Based Therapeutics

MiRNA-based therapies aim to either restore the expression of downregulated miRNAs using miRNAs mimics or inhibit the expression of upregulated miRNAs using miRNA inhibitors [218]. However, the clinical application of these molecules faces several limitations that reduce their efficacy. First, nucleases in the extracellular space rapidly degrade free miRNA mimics and inhibitors and they are quickly excreted by the kidneys, making them unstable; therefore, chemical modifications are often required to enhance their resistance to degradation [219,220]. Second, the negatively charged nature of miRNAs and poor blood perfusion hinder their cellular uptake. To address this, miRNAs are frequently encapsulated into biocompatible delivery systems, such as nanoparticles, which facilitate membrane penetration, protect them from nuclease activity and enhance targeted delivery [220,221]. Third, because a single miRNA can regulate multiple genes and signaling pathways, off-target effects may occur, potentially leading to systemic cytotoxicity, which can be mitigated through the use of nanoparticle-based carriers or exosomes with specific ligands or antibodies, which improve target specificity while reducing toxicity [222,223,224]. Finally, administration of miRNA mimics can activate Toll-like receptors (TRL3, TRL7 and TRL8), triggering immune responses, but chemical modifications can help minimize such recognition and lower the risk of immune activation [220].

However, significant efforts have been made to overcome these limitations. Currently, both viral and non-viral vectors are being explored for miRNA delivery to efficiently transfect the desired oligonucleotide into specific target cells. Non-viral systems offer a biocompatible and less immunogenic alternative while ensuring miRNA stability. These include lipid-based nanocarriers, polymer-based nanoparticles and extracellular vesicles [225]. Among them, lipid-based nanocarriers are the most extensively studied due to their excellent biocompatibility, bioavailability and structural flexibility, which allow surface modifications to improve miRNA encapsulation and release. When designing these delivery systems, factors such as size, composition and geometry are critical, as interactions between nanoparticles and blood components may lead to aggregate formations, reducing delivery efficiency and potentially inducing immune toxicity. To mitigate these effects and enhance systemic circulation and cellular uptake, nanoparticles are often conjugated with polyethylene glycol (PEG), which provides steric stabilization and increases delivery efficiency or with cholesterol or dioleoylphosphatidyl ethanolamine to minimize their toxicity [225,226,227,228]. Polymer-based nanoparticles are composed of natural or synthetic polymers such as poly(lactic-co-glycolic acid) (PLGA), polyethylenimine (PEI), and chitosan, that can be engineered to control premature release and enable sustained miRNA delivery. PLGA nanoparticles exhibit a slow degradation profile and low inflammatory potential, but their opsonization in circulation and limited biocompatibility can restrict therapeutic efficiency. To overcome these issues, incorporating additional positively charged polymers can enhance miRNA encapsulation, reduce toxicity and extend circulation time. Additionally, miRNAs are successfully incorporated into PLGA nanoparticles by encapsulation or by absorption through an electrostatic force. PEI, often regarded as the “gold standard” polymer for miRNA transfection due to its ability to promote endosomal escape, is limited by its low degradability, which may result in polymer accumulation and, consequent, systemic toxicity associated with its molecular weight [228,229,230,231]. Conversely, chitosan-based nanoparticles demonstrate low toxicity, low immunogenicity and high biocompatibility, increasing miRNA encapsulation efficiency. Their degradation is triggered by the acidic environment of tumor cells, allowing for efficient, sustained and controlled miRNA release, which renders this polymer an increase application in both in vitro and in vivo miRNA delivery studies [232,233]. Furthermore, extracellular vesicles have gained attention as natural nanocarriers for miRNA delivery due to their intrinsic biocompatibility, ability to cross biological barriers and stability in circulation. Their lipidic bilayer protects miRNAs from extracellular degradation, while their surface proteins facilitate cellular uptake by target cells, providing a natural mechanism for target delivery. MiRNAs can be incorporated into these vesicles through electroporation, passive incubation or by overexpressing the desired miRNA in parent cells. Compared to lipid- and polymer-based nanocarriers, extracellular vesicles display lower immunogenicity and cytotoxicity, making them a promising therapeutic strategy for miRNA delivery [234,235,236].

Several clinical trials have evaluated miRNA-based therapeutics in cancer, illustrating both their therapeutic potential and the challenges associated with their delivery and safety. A clinical trial (NCT01829971) [237] tested the efficacy of a miR-34a mimic encapsulated in a liposomal nanoparticle in patients with solid tumors, which was administered intravenously. This study, with a two-year duration, showed limited efficacy, with no complete responses and only a few partial responses after five treatment cycles, but resulted in several serious immune-mediated adverse effects and four deaths, leading to its termination. Another clinical trial (NCT02369198) [238] evaluated the safety of administrating a miR-16 mimic encapsulated in bacterial minicells (EnGeneIC Dream Vectors) targeting EGFR in patients with malignant pleural mesothelioma. Results indicated that a dose of 5 × 10^9^ TargomiRs once a week was the maximum tolerable level, with higher or more frequent dosing leading to toxicity despite co-administration with dexamethasone prophylaxis. More recently, 1B3, a synthetic miR-193a-3p mimic, was tested across various tumor types. Its overexpression reduced cell number and proliferation by inducing apoptosis and G0/G1 cell cycle arrest. Following these findings, encapsulation of 1B3 in a lipid nanoparticle delivery system (INT-1B3) demonstrated significant anti-tumor activity through tumor size reduction [239]. Furthermore, INT-1B3’s anti-tumorigenic effect was shown to result from tumor targeting that activates a T-cell-mediated immune response while reducing cytotoxicity [240]. These preclinical results have led to the First-in-Human clinical trial (NCT04675996) to evaluate the safety, pharmacokinetics, pharmacodynamics, and preliminary efficacy of INT-1B3 in the treatment of patients with advanced solid tumors, but the trial was terminated prematurely due to insufficient funding, before meaningful results could be obtained [241]. Taking these findings into consideration, the development of miRNA-based therapy requires careful anticipation and monitoring of immune-mediated toxicities, which have been observed in previous clinical trials. In the specific context of HER2-low breast cancer, the presence of tumor heterogeneity should be acknowledged, as it may contribute to partial treatment responses and the low expression of HER2 requires a delivery system that does not rely exclusively on HER2-mediated uptake, such as lipid nanoparticles or exosomes. These systems can enhance the stability and bioavailability of miRNA and reduce off-target toxicity. Such an approach could combine precise delivery with potent gene-regulatory effects, ultimately improving therapeutic efficacy in HER2-low breast cancer.

### 6.2. MicroRNAs as a Personalized Therapeutic Strategy for Breast Cancer

MiRNAs have been investigated in combination with approved targeted therapies for breast cancer, mainly as modulators of drug resistance and sensitivity. Their ability to regulate key signaling pathways and gene expression networks positions them as promising agents to enhance treatment efficacy and overcome therapy resistance.

Several studies have demonstrated miRNAs as crucial regulators of sensitivity to tamoxifen in ER-positive breast cancer. Bin et al. [242] investigated whether miR-205 could regulate tamoxifen resistance in breast cancer and indeed showed that downregulation of miR-205 in MCF-7 cells increased the expression of its target gene MED1, leading to tamoxifen resistance, whereas the restoration of miR-205 reduced proliferation and resensitized cells to tamoxifen. Similarly, Yue et al. [243] demonstrated that upregulation of miR-190 enhanced sensitivity to tamoxifen by targeting SOX9, which was further validated in vivo with nude mice, where elevated miR-190 levels reduced tumor weight. Conversely, elevated levels of ZEB1 promotes CpG methylation and histone deacetylation of the MIR497HG promoter, silencing miR-195 and miR-497, thereby activating the PI3K/Akt pathway and leading to tamoxifen resistance in ER-positive breast cancer [244]. Moreover, upregulation of miR-221 has been significantly correlated with tamoxifen resistance and disease recurrence in ER-positive breast cancer [142], while upregulation of miR-93 and miR-382-3p and downregulation of miR-182-3p have been linked to tamoxifen resistance. The combined expression profile of these miRNAs demonstrates high predictive accuracy for therapy resistance, with a specificity of 85.7% and a sensitivity of 80.8% in ER-positive breast cancer [245].

In HER2-positive breast cancer, multiple miRNAs have been associated with resistance or sensitivity to anti-HER2 therapies. Rau et al. [246] reported that the downregulation of miR-126 promoted trastuzumab resistance in SKBR3 and BT-474 cells by increasing the expression of PIK3R2. Conversely, it was demonstrated that transfecting these cells with miR-126 mimic restored trastuzumab sensitivity, enhancing its efficacy. Similarly, upregulation of miR-21 promotes trastuzumab resistance by targeting PTEN, activating the PI3K/Akt pathway, and promoting EMT, whereas inhibition of miR-21 expression using antisense oligonucleotides can restore trastuzumab sensitivity [170,247]. Furthermore, Hailin et al. [248] found that miR-200c is frequently downregulated in HER2-positive breast cancer, but its overexpression suppresses tumor sphere formation in vitro and reduces tumor growth and lung metastases in vivo. Other miRNAs exert opposite effects, enhancing sensitivity. Overexpression of miR-33b reduces EMT, proliferation, invasion and migration while inducing apoptosis [249]. The combination of miR-770-5p with trastuzumab blocks Akt and ERK signaling and decreases cell proliferation and invasion [170]. Furthermore, co-transfection of miR-26a and miR-30b mimics also sensitize HER2-positive breast cancer cells to trastuzumab treatment by silencing cyclin *E2* gene [250]. Restoring miR-489 expression inhibits proliferation and tumor growth by targeting SHP2 and HER2, simultaneously suppressing ERK signaling [251], whereas miR-16 upregulation induced by trastuzumab impairs the growth of trastuzumab and lapatinib-resistant cells [252]. Furthermore, combining miR-101-5p mimics with trastuzumab plus lapatinib significantly reduces cell proliferation and induces apoptosis, helping sensitize cells to therapy [35]. In addition, Zhou et al. found that miR-4728 directly targets ErbB3-binding protein 1 (EBP1), reducing HER2 overexpression, and other study found that miR-4728 suppresses ESR1 expression, thereby decreasing sensitivity to hormonal therapy but enhancing the efficacy of anti-HER2 therapies [253,254].

The IGF2/IGF-1R/IRS1 pathway is another crucial regulator of trastuzumab sensitivity. The activation of FOXO3a, triggered by increased IGF2 expression, is modulated by miR-128-3p, miR-30a and miR-193-5p, which target IRS1 and IGF2, maintaining HER2-positive breast cancer cells responsive to trastuzumab. Dysregulation of this axis contributes to drug resistance [249]. Moreover, several miRNAs act as master regulators of oncogenic signaling. For example, Merve et al. [255] showed that miR-564 upregulation functions as a dual inhibitor of the PI3K/Akt/mTOR pathway in MCF-7 cells and the MAPK pathway in MDA-MB-231 cells, suppressing phosphorylation of Akt and ERK1/2. Increased expression of this miRNA was associated with lower levels of mesenchymal markers and inhibition of migration, invasion capacities and tumor growth. Likewise, overexpression of miR-22 targets SIRT1 to reduce MCF-7 cells viability and induce apoptosis, increasing radiosensitivity by suppressing DNA damage repair mechanisms [256].

In another study, Lisa et al. [257] performed a high-throughput screening and identified that miR-15b-5p, miR-19b-2-5p, miR-26b-3p, miR-29a-3p, miR-29c-3p, miR-32-3p, miR-93-3p, miR-101-5p, miR-106a-5p, miR-132-3p, miR-153-3p, miR-518a-5p, miR-744-3p and miR-1237-3p as mimics and miR-361-5p and miR-502-3p as inhibitors combined with trastuzumab and lapatinib significantly decreased HER2-positive cells viability by more than 75%, underscoring the potential of miRNAs to enhance therapeutic efficacy. Similarly, altered expression of miR-23b-3p, miR-195-5p, miR-340-5p and miR-656-5p modulates key targets such as AKT3, CCND1, FDF2, RAF1, MYB, c-MET and PTEN, leading to trastuzumab resistance in HER2-positive breast cancer [258].

An additional study [259] revealed that a triple combination of neratinib plus a CDK4/6 inhibitor plus endocrine therapy was more efficient in reducing proliferation and colony formation in HR-positive/HER2-low breast tumor cells (ZR-75-1) than CDK4/6 inhibitor plus endocrine therapy alone. Notably, the co-administration of a miR-23a-5p mimic could significantly enhance the efficacy of this triple therapy by suppressing HER2 signaling.

To date, no clinical trials have validated the efficacy of combining miRNA-based therapies with approved treatment for breast cancer, which may be due to the challenges inherent to miRNA delivery. Nevertheless, miRNAs have consistently demonstrated a synergistic effect with existing therapies, enhancing their effectiveness, reducing resistance and paving the way for new strategies within the framework of precision medicine.

## 7. Conclusions and Future Perspectives

HER2-low breast cancer is defined as tumors with IHC 1+ or 2+ with negative ISH according to the 2023 ASCO/CAP guidelines. Both ASCO/CAP and ESMO recognize HER2-low as a heterogenous group of tumors, stratified by HR status, with differences at the molecular and genomic levels. In this context, HER2-low/HR-positive tumors display luminal-like molecular profiles, while HER2-low/HR-negative resemble TNBC with characteristic mutations of this subtype. Understanding the mechanisms underlying this heterogeneity is crucial for developing more effective therapeutic strategies.

MiRNAs play a central role in modulating key cellular processes, and their dysregulation in cancer contributes to the activation of distinct hallmarks of malignancy, such as proliferation, apoptosis, invasion and therapy resistance. These molecules can serve as diagnostic, prognostic and predictive biomarkers and their expression may vary according to the heterogeneity of HER2-low tumors. Inter- and intratumor differences may drive distinct miRNA profiles, potentially influencing HR expression; therefore, single-cell analysis, combined with multi-omics, can offer the opportunity to explore the molecular characteristics at the cellular level, enabling the identification of dysregulated miRNA panels that may underline the divergence between HER2-low/HR-positive and HER2-low/HR-negative tumors.

Current HER2-targeted therapies alone provide limited clinical benefit in HER2-low breast cancer. Studies suggest that combining endocrine therapy to target HR with HER2-target agents may improve outcomes, but this strategy remains insufficient in some cases. Integrating miRNA-based therapies with endocrine treatment and anti-HER2 targeted regimens may offer a promising path forward and may provide a triple combination approach to more effectively address HER2-low tumor’s heterogeneity and overcome therapy resistance.

Despite extensive research on breast cancer, to the best of our knowledge, this is the first review highlighting miRNAs as potential regulators of the molecular mechanisms underlying HR expression and as contributors to novel targeted therapeutic strategies for HER2-low tumors. However, further studies are needed to identify specific miRNA signatures that distinctly characterize HER2-low tumors and to elucidate the molecular pathways driving their heterogeneity. Moreover, investigating whether circulating miRNAs can serve as minimally invasive biomarkers for HER2-low disease monitoring could provide valuable insights into tumor progression and treatment response. Integrating these findings may guide the development of novel combination therapies and reshape treatment paradigms toward more personalized care for patients with HER2-low breast cancer.

## Figures and Tables

**Table 1:** Summary of miRNAs that affect cancer hallmarks, demonstrating how they are interconnecting.

Hallmark	miRNA	Main Target	Effect	Ref.
Sustained Proliferative Signaling + Resistance to cell death	miR-203a	PIK3CA; Wnt2b	Upregulated in BC; Inhibits PI3K/Akt and Wnt/β-catenin pathways by reducing PIK3CA and Wnt2b expression to induce cell cycle arrest and decrease proliferation, and promotes Bax expression, increasing the rate of apoptosis	[101]
Activating invasion and metastases	miR-199a-3p	DDR2	Downregulated in GC; Promotes EMT via NFATc1/SOX2/Snai1, autophagy and DNA damage in CSCs, and promotes proliferation, invasion and metastasis by upregulating DDR2	[102]
Sustained proliferative signaling + Activating invasion and metastases	miR-30c-5p	PIK3CA	Downregulated in ESCC; Activates PI3K/Akt pathway by upregulating PIK3CA to increase proliferation, invasion and migration	[103]
Sustained proliferative signaling + Evading growth suppressors + Resistance to cell death + Reprogramming of energy metabolism	miR-122-5p	p53	Upregulated NSCLC; Induces cell proliferation and migration and decreases apoptosis by decreasing p53, and activates MVA pathway	[104]
Sustained proliferative signaling + Evading growth suppressors + Resistance to cell death + Activating Invasion and Metastases	miR-19-3p	PTEN	Upregulated in cervical cancer; Increases proliferation and invasion by inhibiting PTEN, which activates PI3K/Akt pathway and reduces autophagy and early apoptosis	[105]
Sustained proliferative signaling + Resistance to cell death	miR-1246 (extracellular)	DR5	Upregulated in lung cancer; Promotes radioresistance and decreases radiosensitivity, contributing to cell proliferation by reducing DR5 expression	[106]
Sustained proliferative signaling + Resistance to cell death + Activating invasion and metastases	miR-140-3p	BCl-2	Downregulated in GC; Enhances proliferation, migration and invasion, while reducing apoptosis by inhibiting BCL2/BECN1-dependent autophagy	[107]
Sustained proliferative signaling + Evading Growth Suppressors + Resistance to cell death	miR-363-3p	PTEN	Upregulated in BC; Activates the PI3K/Akt signaling pathway by downregulating PTEN, which increases cell proliferation and tamoxifen resistance	[108]
Sustained proliferative signaling + Evading growth suppressors + Enabling replicative immortality	miR-21	PTEN	Upregulated in CRC; Promotes the activation of ERK1/2 pathway by reducing PTEN to increase hTERT expression and maintain telomere length	[109]
Sustained proliferative signaling + Evading growth suppressors + Enabling replicative immortality	miR-19b	PITX1	Upregulated in melanoma; Reactivates hTERT activity and promotes proliferation by inhibiting PITX1 expression	[110]
Activating angiogenesis + Activating invasion and metastasis	miR-6084	ANGPTL4	Upregulated in CRC; Under normal conditions, it inactivates the JAK2/STAT3 signaling pathway by downregulating ANGPTL4, which reduces angiogenesis; under hypoxia, it increases HIF1A expression causing SP1 degradation, thereby enhancing angiogenesis and leading to liver metastasis	[111]
Sustained proliferative signaling + Activating angiogenesis + Activating invasion and metastasis	miR-197-3p	TIMP2/3	Upregulated in metastatic LUAD; Promotes proliferation, migration and tube formation by downregulating TIMP2/3, thereby enhancing VEGFR2 expression, ERK signaling, MMP1 and MT1-MMP expressions.	[112]
Sustained proliferative signaling + Activating invasion and metastasis	miR-34c-5p	MMP2	Downregulated in renal cell carcinoma; MMP2-AS1 sponges this miRNA, which prevents MMP2 repression, thereby enhancing proliferation, migration, EMT and invasion	[113]
Resistance to cell death + Reprogramming energy metabolism	miR-22-3p	GLUT1	Downregulated in HCC; Promotes migration and EMT markers’ expression and reduces apoptosis; Increases GLUT1 expression to increase glucose uptake and therapeutic resistance to sorafenib	[114]
Sustained proliferative signaling + Activating invasion and metastasis + Reprogramming energy metabolism	miR-365-3p	CPT1A	Downregulated in lung cancer; Promotes FAO pathway activation, thereby increasing ATP production and enhances proliferation and migration by upregulating CPT1A	[115]
Evading immune destruction	miR-124	STAT3	Downregulated in GBM; When it is upregulated, it inhibits STAT3 that reduces cytokine and chemokine expressions and increases IL-2, TNF-α and IFN-γ expression, thereby reducing the immunosuppression response	[116]
Evading immune destruction + Tumor-promoting inflammation	miR-27a-3p	MAGI2	Upregulated in BC; It reduces MAGI2 expression, which decreases PTEN and activates PI3K/Akt pathway, promoting PD-L1 expression on macrophages and reduces CD8^+^ T cells	[117]
Activating angiogenesis + Activating invasion and metastases	miR-145	N-RAS	Downregulated in BC; It targets N-RAS/VEGF-A, which increases angiogenesis and promotes cell invasion	[118]
Tumor-promoting inflammation	miR-146a	RIPK2	Downregulated in CRC; It upregulates RIPK2, which stimulates IL-17 production by myeloid cells	[119]
Resistance to cell death + Genome instability and mutations	miR-96	RAD51D; REV1	Upregulated in BC and OC; It reduces RAD51D to decrease the activity of HR repair and targets REV1 to increase chemosensitivity	[120]
Resistance to cell death + Genome instability and mutations	miR-101	DNA-PKcs; ATM	Upregulated in GBM; It targets DNA-PKcs and ATM to impair the DNA DBS repair mechanism, which increases cells sensitivity to irradiation	[121]
Evading immune destruction + Genome instability and mutations	miR-148a-3p	PD-L1	Upregulated in CRC; It targets PD-L1 to stimulate IFN-γ production to the TME, which is indirectly correlated with MMR deficiency, increasing genome instability	[122]

BC: Breast Cancer; BCL-2: B-cell Lymphoma 2; BECN1: Beclin 1; CPT1A: Carnitine Palmitoyltransferase 1A; CRC: Colorectal Cancer; CSC: Cancer Stem Cell; DDR2: Discoidin domain-containing receptor 2; DNA-PKcs: DNA-PK Catalytic Subunit; DR5: Death Receptor 5; EMT: Epithelial–Mesenchymal Transition; ERK1/2: Extracellular Signal-Regulated Kinase ½; ESCC: Esophageal Squamous Cell Carcinoma; FAO: Fatty Acid Oxidation; GBM: Glioblastoma; GC: Gastric Cancer; GLUT1: Glucose Transporter 1; HCC: Hepatocellular Carcinoma; HIF1A: Hypoxia-Inducible Factor 1-α; HR: Homologous Recombination; hTERT: human Telomerase Reverse Transcriptase; IFN-γ: Interferon-gamma; IL-2: Interleukin-2; JAK2: Janus Kinase 2; LUAD: Lung Adenocarcinoma; MAGI2: Membrane-Associated Guanylate Kinase Inverted 2; MMP1: Matrix Metalloproteinase 1; MMP2: Matrix Metalloproteinase 2; MMP2-AS1: Matrix Metalloproteinase 2 Antisense RNA 1; MMR: Mismatch Repair; MT1-MMP: Membrane-type 1 Matrix Metalloproteinase; MVA: Mevalonate; NFATc1: Nuclear Factor Of Activated T-Cells, Cytoplasmic 1; N-RAS: Neuroblastoma RAS viral Oncogene Homolog; NSCLC: Non-Small Cell Lung Cancer; OC: Ovarian Cancer; PD-L1: Programmed Death-Ligand 1; PIK3CA: Phosphatidylinositol-4,5-bisphosphate 3-kinase Catalytic Subunit α; PITX1: Paired-like Homeodomain Transcription Factor 1; PTEN: Phosphatase and Tensin Homolog; RAD51D: RAD51 Paralog D; RIPK2: Receptor-Interacting Serine/Threonine-Protein Kinase 2; SOX2: SRY-Box Transcription Factor 2; SP1: Specificity Protein 1; STAT3: Signal Transducer and Activator of Transcription 3; TIMP2/3: Tissue Inhibitor of Metalloproteinases 2/3; TME: Tumor Microenvironment; TNF-α: Tumor Necrosis Factor-α; Wnt2b: Wnt Family Member 2B.

## Data Availability

Not applicable.

## Abbreviations

The following abbreviations are used in this manuscript:

ADC	Antibody-Drug Conjugate
ADCC	Antibody-Dependent Cellular Cytotoxicity
AI	Aromatase Inhibitors
Akt	Activated Protein Kinase B
AKT3	Threonine-Protein Kinase
ASCO	American Society of Clinical Oncology
ASPP2	Apoptosis-Stimulating Protein 2
CAP	College of American Pathologists
CCND1	Cyclin D1
CDK	Cyclin-Dependent Kinase
c-MET	MET Proto-Oncogene
CNV	Copy Number Variations
DGCR8	DiGeorge Syndrome Critical Region 8
EBP1	ErbB3-Binding Protein 1
ECM	Extracellular Matrix
EGFR	Epidermal Growth Factor Receptor
EMT	Epithelial–Mesenchymal Transition
ER	Estrogen Receptor
ESMO	European Society for Medical Oncology
FDF2	Fibroblast Growth Factor 2
FGF2	Fibroblast Growth Factor 2
FKBP12	FK506-binding protein 12
FOXO1	Forkhead Box O1
HCV	Hepatitis C Virus
HER2	Human Epidermal Growth Factor Receptor 2
HR	Hormone Receptor
IGF-1R	Insulin-like Growth Factor 1 Receptor
IGF2	Insulin-like Growth Factor 2
IHC	Immunohistochemistry
IL-6	Interleukin-6
IRS1	Insulin Receptor Substrate 1
ISH	In Situ Hybridization
JAK2	Janus Kinase 2
MAPK	Mitogen-Activated Protein Kinase
MED1	Mediator Complex Subunit 1
miRISC	miRNA-induced silencing complex
miRNAs	MicroRNAs
mitomiRs	Mitochondrial MicroRNAs
MMP9	Matrix Metalloproteinase 9
mTOR	mammalian Target of Rapamycin
mTORC	mammalian Target of Rapamycin Complex
MYB	V-Myb Avian Myeloblastosis Viral Oncogene Homolog
OS	Overall Survival
PARN	Poly(A)-specific ribonuclease
PDK1	Phosphoinositide-dependent kinase 1
PEG	Polyethylene Glycol
PEI	Polyethylenimine
PFS	Progression-Free Survival
PI3K	Phosphatidylinositol-3-kinase
PIP2	Phosphatidylinositol-4, 5-biphosphate
PIP3	Phosphatidylinositol-3, 4, 5-triphosphate
PLGA	Poly(Lactic-co-Glycolic Acid)
Pre-miRNA	Precursor MicroRNA
Pri-miRNA	Primary MicroRNA
PTEN	Phosphatase and Tensin Homolog
RAF1	Raf Proto-Oncogene Serine
Rb	Retinoblastoma
SERM	Selective Estrogen Receptor Modulators
SHP2	Src Homology Region 2-Containing Protein Tyrosine Phosphatase-2
SIRT1	Sirtuin 1
SOCS1	Suppressor of Cytokine Signaling 1
SOX9	SRY-Box Transcription Factor 9
STAT3	Signal Transducer and Activator of Transcription 3
TAM	Tumor-Associated Macrophages
T-DM1	Trastuzumab Emtansine
T-DXd	Trastuzumab Deruxtecan
TERT	Telomerase Reverse Transcriptase
TGF-β	Transforming Growth Factor-Beta
TKI	Tyrosine Kinase Inhibitors
TME	Tumor Microenvironment
TNBC	Triple-Negative Breast Cancer
TRL	Toll-like Receptors
TSC1/2	Tuberous Sclerosis Complex 1/2
UTR	Untranslated Region
VEGF	Vascular Endothelial Growth Factor
XPO5	Exportin 5
ZEB1	Zinc Finger E-box-binding Homeobox 1
